# Supplementary figures and images for: Genome-wide identification, expression pattern and interacting protein analysis of INDETERMINATE DOMAIN (IDD) gene family in Phalaenopsis equestris
Source: PeerJ. 2024 Sep 25;12:e18073. doi: 10.7717/peerj.18073 (PMC11438434; doi:10.7717/peerj.18073)

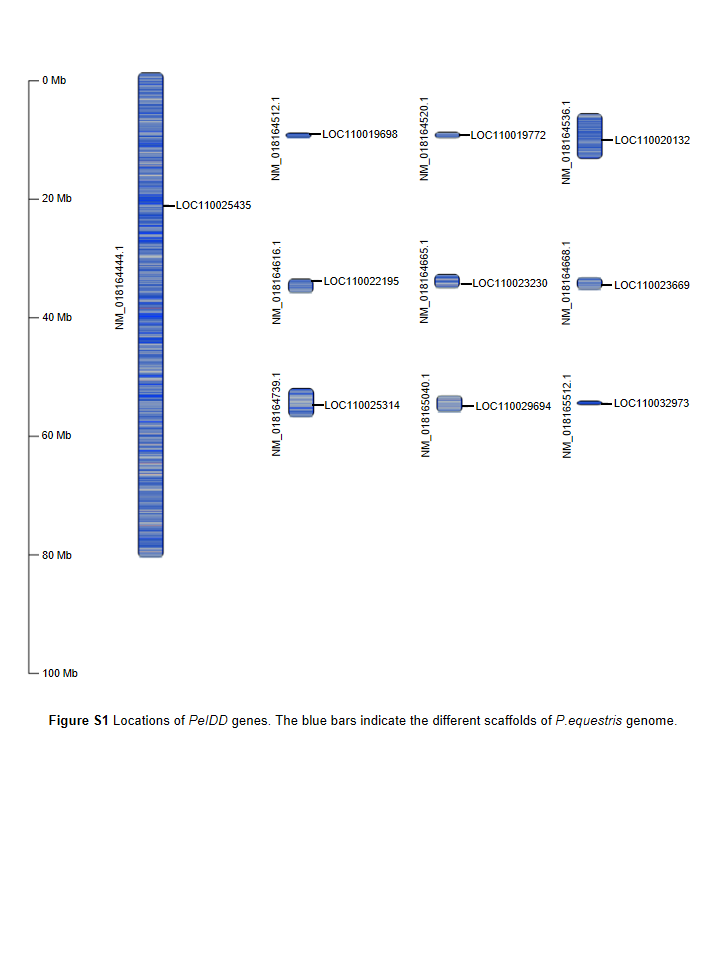

Supplement: Supplemental Information 1 — The blue bars indicate the different scaffolds of P. equestris genome. [file peerj-12-18073-s001.png]

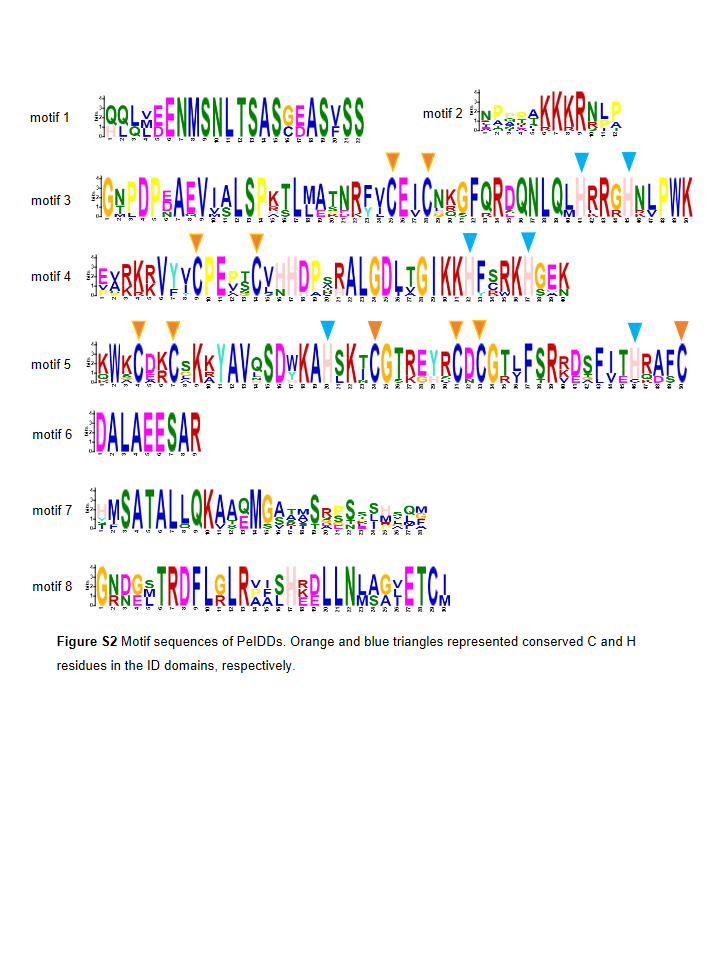

Supplement: Supplemental Information 2 — Orange and blue triangles represented conserved C and H residues in the ID domains, respectively. [file peerj-12-18073-s002.png]
